# The Anatomy of the Large American Finke (Fasciola Magna)

**Published:** 1894-12

**Authors:** Charles Wardell Stiles

**Affiliations:** Zoölogist, Bureau of Animal Industry


### THE ANATOMY OF THE LARGE AMERICAN FLUKE (*FASCIOLA MAGNA*), AND A COMPARISON WITH OTHER SPECIES OF THE GENUS FASCIOLA, S.ST.

By Chas. Wardell Stiles, Ph.D.,
*Zoölogist, Bureau of Animal Industry.*

### CONTAINING ALSO A LIST OF THE CHIEF EPIZOÖTICS OF FASCIOLIASIS (DISTOMATOSIS) AND A BIBLIOGRAPHY OF *FASCIOLA HEPATICA*.

By Albert Hassall, M.R.C.V.S.

(Continued from page 417.)

### Bibliography of F. hepatica.

BY ALBERT HASSALL.

Lutz, A.—
'92.—Zur Lebensgeschichte des Distoma hepaticum ; C. f. B. u. P. XI. No. 25, pp. 783-796.—Rev. in Naturwissenschaftliche Rundschau No. 34 VII. Jahr. 1892, pp. 436-437. (Korschelt.)—Ref. von Lüpke, Rep. der Thierheilk. 10 Heft. Archiv. f. animalische Nahrungsmittelkunde. No. 2, VII. pp. 24-25.
'93.—Weiters zur Lebensgeschichte des Distoma hepaticum; C. f. B. u. P. XIII. No. 10. pp. 320-328.

Lydtin —
('90.)—Ueber die Distomatosis der Haussäug.; Archiv. f. wiss. u. prakt. Thierheilk., xvi, p. 372.

Macé, E.—
('82.)—Recherches anatomiques sur la grande douve du foie (Distoma hepaticum), Paris.

Mackh —
('89.)—Leberegel in der Lunge einer Kuh ; Repert. d. Thierheilk., p. 308.

Mangin —
('32.)—Mém. sur la cachexie aqueuse (pourriture) des bêtes à grosses cornes ; Rec. de Méd. Vétér. Prat. p. 420.

Marshall, J. T.—
('83.)—On a parasite of Limnea truncatula ; Journ. of Conchology. IV. p. 10.

MARSHALL & HURST —
'87.—The Liver fluke of the Sheep; A Junior Course of Practical Zoology. Lond., pp. 25-35. figs. 6-12.

MÉGNIN, P.—
('82.)—Tubercules des poumons chez une Vache causés par des Douves (*Distoma hepaticum*); Comp. rend. de la Soc. de Biol., p. 221.

MEHLIS, Carl. Fr. Ludov.
('25.)—Observationes anatomicæ de Distomate hepatico et lanceolato ad Entozoorum humani Corporis historiam naturalem illustrandum. Acced. tab. aen. color I. Fol. Maj. Gottingæ.

MINOT, C. S.—
('84.)—The Life history of the distoma hepaticum· Boston Med. & Surg. Journ. CX. 418.

MIRAM —
('40.)—Bull. de la Soc. Imp. des Natural. de Moscow, p. 157.

MOJKOWSKI —
('88.)—Przeglad Weterinarsky.
('88.)—Œsterr. Monat. f. Thierheilk. p. 118.

MOLIN —
('59.)—Wiener Sitz. XXXVII. p. 827.

MOROT, CH.—
('87.)—Études statistiques sur la distomatose pulmonaire des Bovidés; Bull. Soc. centr. de Méd. Vétér., p. 38 et 64.
('90.)—Concrétion bronchique volumineuse due à la présence d'une douve dans le Poumon gauche d'une Vache; Bull. d. l Soc. Cent. de Méd. Vét. pp. 407–408.

MORTON, W. J. T.—
'39.—On the Entozoa affecting domesticated animals, and particularly on *F. hepatica*, or Liver Fluke in Sheep; Veterinarian, Vol. XII. pp. 735-738.

MOULINIÉ, J. J.—
('55.)—De la reproduction chez les Trematodes endo-parasites; Mém. de l'institut Genevoise III.

MURRAY, A. J.—
'82.—*Distoma hepaticum*, infesting lungs of cattle; Am. Vet. Rev., p. 100.

NEUMANN, L. G.—
'88.--Traité des Maladies parasitaires, &c. Paris. p. 507-564. fig. 229-238.
'92.—2d Edit. pp. 504-528, figs. 271-281. Paris.
'92.—Translation by Fleming, pp. 517-543, figs. 271-281. London.

OGANESYANTZ, G. Y.—
('73-'74.)—Dvurom pechenochnii v' Alexandropolskom uaizdai (*Distoma hepaticum* in the district of Alexandropolis); Protok. zasaid. Kavkazsk. Med. Obsh., Tiflis. X 205.

OKEN —
'35.—*D. Hepaticum*; Allgem. Naturg. f. alle Stände. Stuttgart, pp. 550-551. Vol. II.

OLFERS —
('16.)—*Distoma hepaticum*; Commen. d. Veget. et anim. Corp. Berl. t. I. p. 4.

OLSSON, P.—
('76.)—Skandinav. Helminthfauna, p. 13.

OTTO, A. W.—
('16.)—Ueber das Nervensystem der Eingeweidewürmer; Mag. d. Gesells. naturf. Fr. zu Berlin. VII.

OWEN, R.—
'43.—Distoma hepaticum ; Lectures on the Comp. Anat. & Phys. of the inv. Animals. London, p. 56, fig. 27.
'55.—2d Edit. p. 80-81, fig. 36.

PARONA —
'87.—Annali Museo Civico. Genova, p. 489.

PERRONCITO, E.—
('81.)—L'anemia dei contadini, fornaciai e minatori in rapporto coll' attuale epidemia negli operai del Gottardo ; Annali d. R. Accad. d'agric. di Torino XXIII.
('84.)—Action du chlorure de sodium sur les Cercaires et de leur desséchment ; Arch. ital. d. Biol. VI. p. 154.
('85.)—Réc. de Méd. Vétér. p. 208.
'86.—*Distoma hepaticum ;* Trattato Teorico-pratico sulle malattie più comuni degli Animali Domestici. Torino. p. 247-249. fig. 95-99.
('87.)—Il medico veterinario. p. 97.

PIANA, G. P.—
('82.)—Le cercarie nei Molluschi studiate in rapporto colla presenza del Distoma epatico e del Distoma lanceolata nel fegato dei Ruminanti domestici ; La clinica Veter. V.

PRUNAC, A.—
('78.)—De la douve ou distome hépatique chez l'homme ; Gaz. d. hôp, Paris, 1147-1149.
('79.)—Ann. Soc. d. Méd. d. Lyon, 2. s., XXVII, 99-109.
('84.)—Note sur la grande Douve du foie (*Distoma hepaticum*) Montpellier et Paris, in 8°. de 14 p.

RAILLIET, A. et Morot—
('85.)—*Distomum hepaticum;* Bull. Soc. Cent. de Méd. Vét. p. 285.

RAILLIST—
'86.—*Distoma hepaticum ;* Éléments de Zoologie medicale et agricole Paris. p. 286 293. fig. 180-189.
'87.—Distomatose du Lapin domestique ; Bull. d. l. Soc. Centr. de Méd. Vét. p. 324.
('90.)—Bull. Soc. Zool. d. France. XV. p. 88.
('93-'94.)—Traité d. Zool. méd. et agric.

RAMDOHR, K. A.—
('14.)—Anat. Bemerk. über d. Egel in der Schafleber ; Mag. d. Ges. Naturf. Freunde zu Berlin, VI. pp. 128-131. Taf. III.

RAYNAUD —
'59.—"A Word on the Cachexia, or Rot in Ruminants." Trans. from the Journ. des Vét. by W. Ernes ; Veterinarian Vol. XXXIII. p. 488.
('60.)—Journ. des Vétér. du midi, p. 222.

REYNÉS, P.—
('69.)—Des moyens de recherches á employer pour retrouver l'origine probable des douves (Distoma hepaticum et Distoma lanceolatum) ; Marseille Méd., VI., 30-36.

RIVOLTA —
('68.)—Nodi nel pulmone dei bovini pro dotti da distomi; Il medico veter., p. 267.
('81.)—*Distomum hepaticum;* Bull. Soc. Cent. de France, p. 68.

REYNAL —

('56.)—Art. Cachexie; Nouv. Dict. prat. de Méd. Vét. Paris.

ROELL —
('69.)—Manuel de Pathologie. Vienne.

ROLLESTON, G.—
('80.)—Note on the Geographical Distribution of Limax agrestis, Arion hortensis & Fasciola hepatica ; Zool. Anz. III, p. 400.
('80.)—The "Times" April 14.

RUDOLPHI, C. A.—
('03.)—Wied. Archiv. III. p. 1. 62.
'09.—Ent. sive Vermium intest. Hist. Nat.—Amstelædami, t II. I. p. 352.
'19.—Ent. Syn. pp. 92, 362-364, 583, 588, 616, & 617.

SAGARRA, V.—
('90.)—Un Caso distoma hepatico en el hombre ; Revista de Med. y cirur. prac. XIV. pp. 505-512.

SCHAPER, A.—
('89.)—Deutsche Zeit. f. Thiermed. XVI. p. 1.

SCHAUINSLAND, H.—
('82.)—Beiträge z. Kenntniss der Embryonalentwickelung der Distomum ; Zool. Anz. v. p. 494.
('83.)—Jenaische Zeitschr. XVI. p. 465.

SCHELL —
('55-'56.)—Mittheil. aus d. thierärztl. Praxis. Berlin.

SCHMIDT—
('87.)—*Distoma hepaticum* in der Lunge eines Rindes ; Arch. f. wiss. u. prakt. Thierheil. XIII. p. 361.

SCHUBART —
('53.)—Over *Distoma hepaticum ;* Aantecken Utrecht Genootsch sectie Natuur. en Geneesk. Ap. p. 28-31.

SCHWARZE, W.—
('86.)—Die postrembryonale Entwicklung. der Tremat; Z. f. w. Z. XLIII. p. 41.

SIEBOLD —
('36.)—Wiegmann's Archiv. p. 217.
('36.)—Müller's Archiv. p. 233.
('45.)—Wiegmann's Archiv. II. 223.

SIMONDS, J. B.—
'61.—Lecture on the Nature and Causes of the Disease known as Rot in Sheep ; The Veterinarian. Vol. XXXIV., p. 274.
('62.)—The Rot in Sheep, its Nature, Cause, Treatment, and Prevention. London.

SMITH, WM. A.—
'63 —On Human Entozoa. Lond. p. 35-36, fig. 7.

Sonsino, P.—

(84a.)—La Fasciola epatica e il suo ciclo vitale ; La Natura, N 32.

(84b.)—Contro lo sviluppo della Fasciola epatica nelle gregge ; La Natura, N 47.

('90.)—Proc. Verb. d. Soc. Tosc. di Sci. Nat. 4 Maggio (*D. caviæ-F. hepatica*).

Sommer, F.—

('80.)—Die Anat. des Leberegels, *Distomum hepaticum*, L.; Z. f. w. Z., XXXIV. p. 539-640.

Spengel, J. W.—

('92.)—Abnormitäten bei *Distomum hepaticum ;* Verhand. de deutsch. Zool. Ges. p. 146-147.

Steel, J. H.—

'81.—*F. Hepatica ;* in A Treatise on the Diseases of the Ox ; London. pp. 93-95. fig. 16. p. 204.

'90.—Rot ; in A Treatise on the Diseases of the Sheep. London, pp, 135-172. figs. 29-48.

Stieda, L.—

('67.)—Anatomie der Plattwürmer. Berlin, 1867. Arch. f. Anat. û. Physiol. p. 52-59, Taf. II.

Stiles, [Charles] Wardell —

'94.—The Anat. of the Large American Fluke (*F. magna*), and a comparison with other species of the genus *Fasciola*, s.st.; vide Journ. Comp. Med. and Vet. Arch. pp.

Stossich, M.—

('91.)—Bull. Soc. Adr. di Sc. Nat. Trieste XIII. p. 111.

'92.—I distomi dei Mammiferi, Trieste. p. 7 ; Programma della civica Scuola Reale superiore.

Willemoes Suhm R. von.—

'73.—Helminthologische Notizen, III: Bau und Embryologie der Trematoden ; Z. f. w. Z., XXIII. p. 332.

Thomas, A. P.—

'81.—First Report of Experiments ; Journ. of Roy. Agric. Soc. of Eng. XVII. p. 19.

('82a.)—Second Report of Experiments; Journ. of Roy. Agric. Soc. of Eng. XVIII. p. II. Lond. p. 439-455. fig. 1-6.

('82b.)—A Parasite of *Limnæa truncatula* ; Journ. of Conchology III. p. 329.

'83a.—The Natural history of the Liver Fluke and the prevention of Rot; Journ. Roy. Agric. Soc. of Eng. XIX. p. 276-305, fig. 1-20.

'83b.—The Veterinarian, 1883. pp. 29, 39, 40, 180 and 469.

'83c.—The Life history of the Liver Fluke; Quart. Journ. of Micros. Soc. No. 89.

Turton, W.—

('07.)—The British fauna, containing a compendium of the Zoology of the British Islands ; arranged according to the Linnean System in- 12. Swansea.

Taiche—

('34.)—Réc. de Méd. Vétér. Prat. p. 289.

Taylor, W.—

('84.)—*Distomata hominis;* China Imp. Customs. Med. Rep. Shanghai, XXVII. 44-54.

'85.—Further note on *Distoma hepaticum*; China Imp. Customs. Med. Rep 1884. Shanghai, 1885, XXVIII. 58-60. 1 map.

Vogt & Yung—

'88.— *Distomum hepaticum*, Lin; Traité D'Anatomie Comparée Pratique. Paris pp. 226-248. figs. 99-108.

Waime—

('62.)—Ann. de méd. vétér. p. 32.

Waldinger—

('18.)—Ueber Würmer in der Lunge und Leber der Schafe. Vienne.

Walter, H.—

('66.)—Ber. f. Naturk. Offenbach. VII. p. 13.

Weinland, D. F.—

('74.)—Archiv f. Naturg. II. p. 423.

('75.)—Die Weichthierfauna der Schwäbischen Alb. Stuttgart, Von p. 101.

('83.)—Zur Entwicklungs. des Leberegels, *Distoma hepaticum*; Jhrshftn. d. Ver. f. Vaterl. Naturk. in Württ, p. 89-98.

Wernicke, R.—

('86.)—Die Parasiten der Haustiere in Buenos Ayres; Deut. Zeit. f. Thiermed-u-vergl. Pathol. XII. p. 304.

Wilson, A.—

'80.—On the occurrence of the common Fluke (*F. hepatica*) in the human subject; Edinb. Med. J., XXV., 413-417.

Wyss, O.—

('68.)—Ein fall von *Distomum hepaticum* beim Menschen; Arch. d. Heilk., Leipz., IX, 172-177, 1 pl.

Zeder, J. H. G.—

('00.)—Nachtrag zu Göze's Naturgeschichte der Eingeweidewürmer.

('03.)—Nat. d. Eingeweidew. 209.

Zündel, A.—

'74.—Cachexie aqueuse; Art. in Dictionnaire de Méd., de Chir., et d'Hyg. vét. par L. H. J. Hurtrel D'Arboval. Ed. par A. Zündel. Paris, Tome I. pp. 224-230. figs. 95-98.

'80.—La distomatose ou cachexie aqueuse du mouton. Strasbourg. pp. 30. (woodcuts.)

Bouley—

Rapport fait à la soc. nat. d'agricult. . . . sur un mém. intitulé; Considerations sur l'étiologie de la distomatose ou cachexie aqueuse des moutons. ib. p. 21-30.

Zürn—

'82.—Die Schmarotzer unserer Haussaügeth. Weimar, p. 207. tab. IV. fig. 5-8.

